# Generation Y medical doctors’ experiences of a positive psychology 2.0 intervention for burnout in a South African public hospital

**DOI:** 10.3389/fpsyg.2022.861872

**Published:** 2022-10-12

**Authors:** Rudolf M. Oosthuizen, Keitumetse Disemelo, Claude-Hélène Mayer

**Affiliations:** ^1^Department of Industrial and Organisational Psychology, University of South Africa, Pretoria, South Africa; ^2^Department of Industrial Psychology and People Management, University of Johannesburg, Johannesburg, South Africa

**Keywords:** burnout, Generation Y, medical doctors, South African public health sector, positive psychology 2.0 intervention, culture-specific research

## Abstract

The aim of the research was to explore experiences of a Positive Psychology 2.0 (PP2.0) intervention for burnout among Generation Y medical doctors working in a South African public hospital. The emphasis was on the potential benefits and recommended intervention amendments in a specific cultural context of South Africa. A phenomenological approach was followed in the collective case study. The Maslach Burnout Inventory was administered in phases I and III to quantify the level of burnout. This study offers findings that could be incorporated into a bigger burnout intervention strategy in the South African public hospital, involving all stakeholders to ensure that burnout is combatted on a long-term basis. Furthermore, the findings emphasized certain culture-specific structural issues and the impact that the neglection of burnout has on newly qualified medical doctors working in a South African public hospital and patients in their care. Certain recommendations were made for the South African public hospital for future research in PP2.0 interventions and for facilitators working with burnout among newly qualified medical doctors.

## Introduction

New models in existential positive psychology (EPP) are needed to capture and promote a complex and systemic understanding of the world and life. Existential positive psychology (Wong, 2009, 2011) is also termed the second wave of positive psychology (PP2.0) (Ivtzan et al., 2015; Lomas and Ivtzan, 2016); and PP2.0 is an expansion of the first wave of positive psychology – PP1.0 (Seligman and Csikszentmihalyi, 2000) – a theory criticized for being extremely fixated on positivity (e.g., Held, 2004; Wong and Roy, 2017). In addition to the positive virtues of human performance offered in the PP1.0 research, PP2.0 asserts that to bring out the best in people, it is essential to include the dark side of life. According to this PP2.0 perspective, heart-breaking experiences, shock, fatality, disease and existential abysses, among other encounters, may intuitively be deemed detrimental to individual and spiritual development (Wong, 2011; Carreno and Pérez-Escobar, 2019; Wong et al., 2021).

PP2.0 recognizes that most people’s lives are lived in negative territory, which is in contrast to PP1.0’s focus on the neutral and positive territories of life. People are harmed or injured on many levels – personally, interpersonally, and societally. Those with different beliefs can be tortured and killed in an authoritarian society. When people are not morally constrained by their conscience, smart people can destroy many lives in pursuit of their own happiness and success aided by wealth and digital power. PP2.0, as conceptualized by Wong (2011), suggests that the most promising method for achieving the mission of PP1.0 is to confront the darker aspects of human existence and understand the unique expressions of well-being in different cultures. PP2.0 emphasized on the one hand the existential universal, while on the other it focused on indigenous cultural expression. To summarize, PP2.0 refers to a new approach to PP1.0 that is more nuanced and balanced. There are two pillars of PP2.0: existential psychology (Wong, 2009, 2016) and indigenous psychology (Wong, 2013; Chang et al., 2016). The combination of these two themes results in positive psychology with greater depth and breadth by including existential perspectives (Jans-Beken and Wong, 2019).

## Burnout

Burnout has been identified as a growing topic in the context of the medical profession (Bahrs, 2019). Burnout was added to the World Health Organizations’ International Disease Classification (ICD-11) as an occupational phenomenon (World Health Organization [WHO], 2019), indicating global prevalence globally, which is a cause for concern. This study contextualized burnout, as described by Maslach and Jackson (1981), and the proposed process model by Leiter and Maslach (1988). Burnout is a three-dimensional construct characterized by increased emotional exhaustion, depersonalization, cynicism, disengagement or detachment; thereby, implying a negative attitude toward several aspects of the job; and an increased tendency for negative evaluation of self as lacking professional accomplishment or competence with reduced feelings of personal job-related efficacy (Maslach and Jackson, 1981, 1986; Maslach, 1982; Maslach et al., 1996). The process model suggests that emotional exhaustion is the preliminary central component that often leads to the other two factors, namely depersonalization and reduced personal accomplishment (Maslach and Jackson, 1981; Leiter and Maslach, 1988). Thereby, emotional exhaustion is explained as the depletion of psychological resources; or the feeling of being strained beyond one’s ability and being depleted of emotional and physical resources (Maslach and Jackson, 1986). Cynicism or depersonalization typically involves negative, pessimistic, insensitive attitudes and a detachment from work that can lead to a dehumanizing view of patients, work and/or workplace relationships (Nuallong, 2013). In extreme cases, this dehumanized view of patients can lead to employees who view patients as undeserving of their services (Maslach et al., 1996). The lack of professional competence, which is the final component in the process model (Leiter and Maslach, 1988), is a negative self-perception, especially in the work context, and an extreme feeling of dissatisfaction about job accomplishments (Maslach et al., 1996). This type of view is indicative of a distorted thinking pattern and an incongruous view of self, which often leads to a lack of involvement in the workplace and having feelings of decreased work performance or the ability to complete tasks (Nuallong, 2013). While burnout is an important aspect connected to the individual, it is at the same time an expression of the socio-cultural context (Bahrs, 2019).

The results the study by Schaufeli et al. (2020) provide initial evidence for a new conceptualization of burnout and an associated measure, the Burnout Assessment Tool (BAT). Specifically, evidence is found for the reliability and factorial and construct validity of the BAT. According to their results the BAT can be seen as a viable, alternative burnout measure, that assesses the burnout syndrome as such (total score), as well as its core components and secondary symptoms. Empirically based burnout interventions are urgently needed due to the negative impact of burnout on at-risk professionals, such as newly qualified medical doctors in South Africa (Mathias and Wentzel, 2017; Discovery Health, 2018; Liebenberg et al., 2018; Hlatshaneni, 2019). Employees in developing countries, such as South Africa, must deal with increased levels of job stress due to the reality of their work environments, which includes a low number of staff in the under-resourced and ailing public hospital (Carod-Artal and Vázquez-Cabrera, 2013; Mathias and Wentzel, 2017; Discovery Health, 2018; Liebenberg et al., 2018; Hlatshaneni, 2019). There are limited empirically based interventions for burnout (Sorenson et al., 2016; Werneburg et al., 2018), even for at-risk populations, such as medical doctors working in a South African public hospital. Burnout has a negative impact on their private lives, the services offered and recipients of care (Discovery Health, 2018; Liebenberg et al., 2018; Hlatshaneni, 2019); yet, most research focuses on identifying the risk factors for medical doctors in the described context and rarely on potential intervention (Sorenson et al., 2016; Werneburg et al., 2018). The medical doctors are mainly in the Generation Y cohort in South Africa aged between 25 and 35 (Generation project, 2018; Kane, 2019). They are exposed to various demanding and traumatic circumstances such as poor resources; an increased number of patients that the public hospital is meant to serve; appalling infrastructure; and high cases of trauma and/or severe chronic and untreatable diseases (HIV/AIDS, cancer and resistant TB), which are a common reality in South Africa (Phalime, 2014; Mathias and Wentzel, 2017; Hlatshaneni, 2019), resulting in increasing patient deaths, a higher burden of disease (Liebenberg et al., 2018) and unjust prejudiced working conditions with illegal labor practices (Erasmus et al., 2012; Hlatshaneni, 2019). Currently, the COVID-19 pandemic is putting increased pressure on a non-functional South African public health care system, overwhelming Generation Y medical doctors and putting them at a higher risk of burnout (Mbunge, 2020). The public hospital in South Africa is reported to have alarming labor practices such as staff shortages, long shifts with no ability to take adequate leave, reduced mentoring and teaching, and reduced support services (required of their seniors) to help with trauma from the work environment (Erasmus et al., 2012; Liebenberg et al., 2018; Hlatshaneni, 2019) that impair Generation Y medical doctors, their patients and familial and societal systems. They work long hours as they are required to work 30-h shifts with little or no provision for illness (Erasmus et al., 2012; Sirsawy et al., 2016; Discovery Health, 2018; Liebenberg et al., 2018; Hlatshaneni, 2019). These realities add to the workload burden, leading to a complex decision-making process, forcing them to make critical, on-the-spot decisions and dealing with crisis situations continually (Sirsawy et al., 2016; Lemaire and Wallace, 2017; Liebenberg et al., 2018). Combined with other influences such as personality traits, limited working experience, personal and work stressors; and the South African government, politics, society and the economy (Bährer-Kohler, 2013; Lemaire and Wallace, 2017), these factors increase the burnout rate among newly qualified medical doctors working in the public hospital (Mathias and Wentzel, 2017; Discovery Health, 2018; Liebenberg et al., 2018; Hlatshaneni, 2019). Erasmus et al. (2012), (p. 655) labeled Generation Y medical doctors in the South African public health context as “slaves of the state” due to the illegal labor practices they are exposed to as part of their mandatory training to become medical doctors; sadly a similar negative narrative is seen in recent research (Discovery Health, 2018; Hlatshaneni, 2019; Mbunge, 2020). Irrespective of whether the experiences of Generation Y doctors are similar to those of other generations, or completely different, they must be reported; and supportive models must be developed for the South African public hospital to ensure that they do not emigrate or exit the field, but rather stay and grow in the field of medicine.

## Positive psychology 2.0 intervention

PP1.0 highlights the importance of virtues and people’s personal strengths, such as optimism and appreciation (Peterson and Seligman, 2004; Lopez and Snyder, 2009). In contrast, PP2.0 (Wong, 2011, 2019a,^2021^; Wong et al., 2021) accentuate collective essential principles that are vital for endurance and flourishing, such as courage and kindness, specifically during challenging times (Wong, 2020; Chen et al., 2021; Arslan and Wong, 2022), and accountability (Wong, 2008, 2016, 2019b). This is where PP2.0 plays a huge role, as the focus is on shifting one’s perspective. The emphasis of PP2.0 intervention for newly qualified medical doctors was on promoting the shift for them toward adapting and coping; taking control of what they can; and being empowered to care for themselves amid the ongoing challenge that is their compulsory training and internship in the public hospital in South Africa.

A facilitated exploration of experiences falling within the definition of PP2.0, and the choices for understanding and change that this PP2.0 intervention may present, was considered. It employs certain communication skills to support clients in developing different perspectives to their situations; thereby, uncovering new, goal-oriented solutions (Cox, 2013). The aim was not on alleviating dysfunction or distress, but rather on facilitating well-being and favorable adjusted functioning (Joseph, 2006). This is closely related to Rogers’ person-centered approach (1959, 1963), which focuses on facilitation toward better functioning by using a non-judgmental environment and certain skills to promote inherent potential such as congruence, unconditional positive regard, acceptance; and lastly, precise empathetic support (Rogers, 1959, 1963). A person-centered PP2.0 approach was adopted in this study as the facilitator of the focus group sessions applied selected Rogerian attitudes, skills and behaviors in the various PP2.0 intervention phases to establish rapport with the group of medical doctors; to facilitate the gaining of skills toward optimal functioning; and to accumulate indications of their experiences of the intervention.

## Focus of study

The study focused on the at-risk group of newly qualified Generation Y medical doctors working in a public hospital in South Africa and a proposed PP2.0 intervention for burnout. It explored their experiences of the intervention, thereby emphasizing potential benefits and recommended amendments. The aim of the research was to describe the lived experience of a sample of Generation Y medical doctors working in a South African public hospital in a way that increases the understanding of their experience of an intervention for burnout to explore its benefits and applicability. The research question is: How does a sample of South African Generation Y medical doctors working in a South African public hospital understand and experience the PP2.0 intervention to combat burnout?

## Materials and methods

### Research approach

A qualitative phenomenological and humanistic facilitator perspective (Rogers, 1959; Mason, 2002; Finlay, 2008; Christensen et al., 2017; Creswell and Creswell, 2018) was followed to obtain descriptions of the lived phenomenon of the experiences of Generation Y medical doctors with a group PP2.0 intervention for the burnout process.

### Research design

The research design was qualitative using triangulation from an interpretive paradigm (Creswell and Creswell, 2003; Mertens and Hesse-Biber, 2012; Creswell, 2013; Shannon-Baker, 2015; Creswell and Creswell, 2018). The rationale for the triangulation was an attempt to get a full picture of the phenomenon being studied (Kopala and Suzuki, 1999; Shannon-Baker, 2015). The study took on a phenomenological design, using a collective case study method in three phases: phase I – pre-PP2.0 intervention; phase II – PP2.0 intervention; and phase III – post-PP2.0 intervention. The Maslach Burnout Inventory – General Survey (MBI-GS) was administered in phases I and III to quantify the level of burnout; and some questions were incorporated in phase II as part of the semi-structured guiding questions of the burnout intervention.

### Sample

The clinical manager at the hospital responsible for the intern and community service doctors was approached for the study. The clinical manager introduced the researcher – which presented a motivation to 98 participants – to participate. The interested participants were recruited to participate in the study. The purposive sampling approach was used to select the relevant participants in terms of the research question (Babbie and Mouton, 2010). The aim of the study was not to generalize across the larger group, but rather to understand their lived experiences in the group intervention. The selection criteria were as follows: aged 25 to 35, Generation Y (Generation project, 2018; Kane, 2019); a medical degree (MBChB); current employment in the public hospital in South Africa for more than 12 months; and a high level of burnout, as measured by the MBI-GS in phase I. Participants who met all criteria in phase I were eligible to participate in phases II and III of the study. Most participants were from a black South African cultural background, including different ethnic group members, such as Sotho, Venda, Tsonga, Tswana and Pedi.

### Measures

The Maslach Burnout Inventory – General Survey (MBI-GS) was used as part of the data collection process in all three phases of the study. The MBI-GS is a valid and reliable tool to measure burnout, which has been used by researchers in South Africa in several studies with similar samples (Peltzer et al., 2003; Thomas and Valli, 2006). A validation study in South Africa on the BAT (De Beer et al., 2022) was underway during the times of this research, thus the preference of the MBI-GS. It was used in phase I to evaluate and measure the level of burnout objectively, which was the final criterion for selecting participants. In phase II, some MBI-GS questions were adapted and used as semi-structured guiding questions as part of the burnout intervention process, using focus group sessions. The level of burnout post-exposure to the intervention was evaluated in phase III.

### Data collection, analysis and interpretation

The data collection involved administering the Maslach Burnout Inventory – General Survey (MBI-GS) and a designed focus group intervention session for burnout (Moustakas, 1994; Creswell, 2013). An interpretive framework was adopted where the focus of ontological interest was on the experiences and meanings of the sample (Whitley, 2002; Scotland, 2012). An idiographic approach, being an all-inclusive representation that attempts to describe experiences of participants (Maykut and Morehouse, 1994; Beck and Jackson, 2020), was also employed to understand and describe the experiences of Generation Y medical doctors of a burnout intervention process (Maree and Van der Westhuizen, 2007). The unit of analysis, which promotes replication for comparison (Yin, 2018), was the descriptions of the experiences of a group intervention process for burnout among Generation Y medical doctors.

For data interpretation: recorded sessions for phases II and III were transcribed verbatim and analyzed, using the descriptive analysis technique of Tesch (1990). The findings were interpreted in light of the literature review to evaluate support for previous research; highlight new findings; and contextualize them according to the research design (Creswell, 2013, 2016). MBI_GS questionnaires were scored in phase I to determine the levels of burnout for participation and phase III to assess the level of burnout in phase III; and the findings were integrated into the conclusions. Descriptive phenomenological analysis and coding were done for phases II and III to organize the data. Observations were highlighted and bracketing notes revised. Data was re-read to further uncover deeper meanings. The descriptive analysis technique, suggested by Creswell and Creswell (2003) and Creswell (2013) was used; transcription of the interviews followed by the identification of statements related to the participants’ experiences. The actual views and feelings of burnout, as well as the intervention and practical application information gathered, were noted. The relevant information was broken down into smaller phrases that reflected specific thoughts. Statements were grouped into measurement units, reflecting various meanings or aspects of the experience of the intervention as the process proceeded and concluded. Deviating viewpoints were sought regarding the intervention for burnout. Lastly, the focus was on constructing a combination of viewpoints to develop an overall description of the intervention for burnout as the participants experienced it. The data was verified and scrutinized by the participants to ensure that it was a true reflection of their experiences.

### Research procedure

The burnout PP2.0 intervention process among Generation Y medical doctors working in the public hospital in South Africa was completed in three phases. Phase I was the pre-PP2.0 intervention; phase II the PP2.0 intervention; and phase III the post-PP2.0 intervention. Phases II and III made use of focus groups to collect data.

#### Phase I: Pre-PP2.0 intervention

Phase I was aimed at conducting a literature review, and applying for and receiving ethical approval for the research from the academic institution and the public health hospital. It was aimed at screening the potential group of participants for the final criterion of participation – a high level of burnout, as determined by MBI-GS scores. The screening was conducted 2 weeks before the actual implementation of the intervention in phase II. The clinical manager invited 30 Generation Y medical doctors who met the first three initial criteria of participation to a meeting on the researcher’s behalf. The group members included black, white and colored doctors. The clinical manager introduced the researcher and left the room. The study was briefly introduced, and 12 non-interested participants were excused. Eighteen consenting participants from different cultural backgrounds (black and white South Africans) remained and voluntarily completed the MBI-GS for screening purposes for the final criterion of selection.

All 18 Generation Y medical doctors met the final criterion of high levels of burnout. The 18 qualifying and willing participants were randomly listed. All of them were contacted telephonically and invited to participate in the focus group discussions (phases II and III) 2 days after the meeting. All 18 voiced interest in participating in phases II and III. Initially, two groups with nine participants each were envisaged. Once the Generation Y medical doctors showed interest and had committed to attend, they were randomly allocated to a group and given a potential date, time and venue for the first focus group. Although 18 confirmed, only ten participants attended the session for phase II. Reasons given for absence a day before the scheduled focus group included sickness; an emergency that required the doctor to travel home; taking on extra calls for a sick colleague; and one doctor had a sudden event at home that required his/her presence. Only ten participants remained and the decision was taken to have only one focus group on a specific day with available participants. After the difficulties faced in securing a group and dates suitable for all 18 to attend the intervention, the group format and intervention process had to be adjusted to fit the reality.

#### Phase II: PP2.0 intervention

The initial envisaged group intervention for burnout among Generation Y medical doctors, comprising three sessions, had to be adjusted due to the challenges the participants had in taking time off, and the final intervention was a 4.5-h session. Six female and four male participants arrived at the venue; they signed an informed consent form for participation and the session commenced. The participants were reminded of their voluntary participation, and the ethical considerations were explained. The rules of the focus group were described, including anonymity, recording and note-taking. The use of pseudonyms, open communication and anonymity of the content was reiterated.

An ice breaker commenced, then the purpose and format of the sessions were discussed and the participants were informed that the final session (phase III) would be held 6 months after the initial session. The participants’ expectations were presented and questions were posed. The first part of the session focused on discussing the experiences of working as a medical doctor in the public hospital at the hospital. Each participant was given a chance to speak and was requested to state his/her pseudonym before speaking for the first time. The focus group session – which was the adjusted designed intervention for the study – started slowly, but with time the participants seemed to lower their guard; it could be that the focus group was a platform that fit their need for debriefing and that they were comfortable with the approach taken in the intervention.

The initial question posed was:

• “What has been your experience of working at the hospital in the Limpopo Province?”

A discussion of burnout followed and some of the sub-questions posed were:

• How do you understand burnout?
• How was completing the questionnaire?

The definition of burnout and the process model of burnout were discussed. The third discussion point started with stories and personal experiences of burnout. The question posed was:

1. “What has been your experience of burnout?”

The risk factors for burnout were explained and the discussion was summarized.

The following discussion focused on individual experiences of the manifestation of burnout. Each participant was given a chance to speak about his/her experiences and coping strategies. Ways of coping were suggested, including relaxation and breathing exercises. Each participant was given the opportunity to pose questions or comments he/she had.

Table 1 below provides – in detail – the possible guiding questions comprising items from the MBI-GS (Maslach et al., 1996) and the Professional Quality of Life Scale (ProQOL 5) (Stamm, 2009) that considered burnout measures. These were paraphrased in the 4.5-h session and were essential to ensure validity, reliability and easy replication of the intervention process in future studies.

**TABLE 1:** Phase II intervention.

**(1) Emotional exhaustion component guideline:** (1.1) Describe the feelings you experience because of work. Describe how work makes you feel emotionally. How would you describe the feeling at the end of your workday and when you get up in the morning? (1.2) Describe what working with people the entire day does to you. Depending on the answers, the researcher would then follow up with: would you say that working with people puts a strain on you? If yes, how so? There was a short discussion about their understanding of burnout, the three dimensions of burnout Maslach and Jackson (1981), and the proposed process model (Leiter and Maslach, 1988). (1.3) From the explanations and your understanding of burnout, would you say that you feel burnt out from your work? Give a full description of the experience. What is your individual experience of the dimensions, if any? (1.4) How do you feel about your job? Do you feel you have strength to carry on in the environment and circumstances?
**(2) Depersonalization component guideline:** (2.1) Certain coping mechanisms are used by various individuals. Describe the way you find yourself treating patients. (2.2) Looking back to how you were before qualifying, and now, describe what has happened to your feelings toward people? Follow up with the following if needed: do you have concerns about being hardened emotionally?
**(3) Reduced accomplishment or personal inefficiency component guideline:** (3.1) Do you deal with your patients’ problems effectively? (3.2) Think back to your interaction with patients. Are you able to create a relaxed atmosphere with them? What makes it difficult/easier? (3.3) Has there ever been a time when you were able to feel overjoyed after working with patients? If so, are you still able to feel this euphoria now? Describe the worthwhile accomplishments in this job. (3.4) Are there a few emotional problems that you need to deal with in the work environment? How often do they occur? Describe how you deal with them.
**(4) Compassion fatigue and burnout factor guideline:** (4.1) Describe your feelings. Would you say you are generally happy? Would you describe yourself as connected to others? (4.2) Would you describe yourself as a sensitive person? Has it always been this way? Do you feel you are overly sensitive? Have you been told that you are overly sensitive? Give details of your experiences with traumatic cases that you see at work. What sort of cases? How do they impact you? How do you cope? (4.3) What is your experience of the amount of work? What do you think about the system or structure generally at work? (4.4) How do you feel about being a helper (medical doctors) now? How do you feel about your future as a medical doctor?
**(5) Coping strategies and view/perceptions of situations:** (5.1) Describe in detail situation(s) that cause negative (such as burnout) and positive experiences within you that occur in your work environment. What have you done previously to cope with these negative situations that often lead to negative experiences? How do you deal with the work and pressure or events that lead to the negative experiences mentioned? How do you cope? What are the positives and negatives? Suggest any better way of coping. (5.2) What is your view of your ability to cope or deal with the situation(s) that lead to the negative experiences? Describe the support you have for your experiences. There was a discussion regarding negative and alternative coping strategies. Responses were written on flipchart paper, starting with a typical situation/event that often leads to burnout based on the participants; the negative coping strategies that were used before; and possible alternative coping strategies, as discussed in the group. There was also a discussion on why the coping strategies were considered negative, or why they were positive/beneficial. The group reflected on their feelings at the end of the session and a discussion followed of the lessons learnt. A feedback process was conducted to deepen the experiences of the intervention. Participant were asked the following questions: Describe how you are feeling following the session? What have you learnt? What will you embrace and what do you think you will remember the most? To conclude, there was a discussion of an action plan for the weeks prior to phase III; this was done with the hope to have an ongoing process. The needs for the next session were discussed, as well as the venue, time and date. The availability of the facilitator in-between sessions was discussed and the session was ended.

#### Phase III: Post-PP2.0 intervention

Phase III was primarily a feedback discussion conducted 6 months after the intervention for burnout among Generation Y medical doctors, reflecting on their post-PP2.0 intervention experience of work. Only six Generation Y medical doctors were available and able to participate in phase III. The reasons for the absence of some doctors included illness, having to go home unexpectedly and having to be on call unexpectedly due to an ill colleague. The duration of the session was 1.5 h with an additional 30 min allocated for completion of the MBI-GS. The details of the 10 participants in phase II and the six participants in phase III of the study are as shown in Table 2.

**TABLE 2:** Participants in phase II and phase III.

	Age	Race	Gender	Marital status	Phase III participation
Participant 1	25	Black	Female	Single	Yes
Participant 2	25	Black	Female	Single	Yes
Participant 3	27	Black	Female	Single	No
Participant 4	27	Black	Female	Single	No
Participant 5	26	Black	Female	Married	Yes
Participant 6	27	Black	Male	Single	Yes
Participant 7	28	Black	Male	Single	Yes
Participant 8	25	Black	Female	Single	No
Participant 9	27	White	Female	Married	Yes
Participant 10	27	Black	Male	Single	No

Table 3 that follows gives a guideline of questions that were posed in phase III, to the six participants during a 2-h session and potential follow up inquiry to elicit more detail. The questions were paraphrased.

**TABLE 3:** Phase III intervention session.

The participants were given a maximum of 30 min to complete the MBI-GS and to determine objective levels of burnout post the intervention. Following that, a 1.5-h session ensued and participants were asked the following: (1) Did you experience burnout and burnout dimensions following our past session? Describe any negative experiences or challenges at work, in detail. What emotions or feelings did the experiences elicit? How did you cope? (2) Was there the opportunity to attempt to use the coping strategies discussed previously? Any challenges with the coping strategies? How were you feeling after your attempt at coping? How are you feeling now? (3) Looking back, was it the best or better way to cope? Explain. Should you add or remove anything from your coping strategies attempted? (4) What would be the best way to handle challenges going forward to elicit positive coping? (5) What are the views of others in the group? What should the person add/remove from their coping strategies? (6) Describe your feelings. How are you feeling in the morning about going to work? Would you describe yourself as connected to others? Are there any changes following the last session? Elaborate. (7) How do you feel about being a medical doctor now? How do you feel about your future? (8) Each participant was asked the following to evaluate the intervention: • How are you post the intervention? • Describe the experience of the intervention for burnout. What did you struggle with in the intervention? What did it lack? What would you add to or change in the intervention you were exposed to? What would you always remember from the experience? Lessons learnt? Were your expectations met by the intervention? • What have you gathered about burnout and your personal experiences at the workplace? • What is your experience and coping strategies now at work? (9) Conclusion and the way forward. The discussion around burnout and the experiences of the intervention were summarized. The negative and alternative coping strategies to attempt in the future work context were discussed. The group reflected on their feelings and the lessons learnt. Feedback was given to deepen the experience of the intervention. The following questions were asked for participants: Describe how you are feeling following exposure to the intervention for burnout. What have you learnt? What will you take forth and what do you think you will remember the most? To conclude, there was a discussion of possible future stressors and action plan discussions for everyone for future coping strategies. The participants were encouraged to elicit self-management in order to prevent and manage burnout in an attempt to cope. Psychoeducation of available help and resources was done and the session was closed.

### Ethical considerations and quality criteria

Ethical clearance was obtained from a South African academic institution as well as the hospital where the research was conducted. The study was classified as a psychological risk at a low level. An external network of private and public professionals was established by the facilitator comprising several identified professional colleagues in the province in the private/public sector for when interventions were needed on a long-term basis; or for psychological issues that could be evoked in the research process. Each participant had a medical aid and could use the professionals at their own cost; and the public sector professionals offered a free service.

The research was conducted in an ethical manner bounded by the ethical code of psychology in South Africa (Health Professions Council of South Africa [HPCSA], 2022). The following ethical aspects that guide research were considered (Health Professions Council of South Africa [HPCSA], 2022): The participants were granted privacy. The group agreed that the discussion was considered confidential outside the group. Anonymity was maintained as the participants used pseudonyms in the focus groups and their real names are not quoted in the study. All the information was stored safely.

This study employed certain validation strategies, namely credibility, transferability, dependability, confirmability and authentic inquiry (Creswell and Miller, 2000; Creswell and Plano Clark, 2011; Creswell, 2013). These were achieved in the study using methods such as ongoing bracketing throughout the entire process; so the perspectives heard were those of the participants, and not those of the facilitator, to ensure that personal biases were put aside. Comments on past experiences, biases, prejudices and orientations could likely shape the approach and interpretation of the study. The researcher’s positioning in the study is important to note. The study was motivated by the facilitator’s experience of burnout when she studied for her master’s degree in Clinical Psychology at the age of 22. She was expected to see clientele in the South African public hospital. She also had a compulsory internship year and subsequently a mandatory community service year in the same sector. In retrospect, she suffered burnout and its consequences – which manifested as severe emotional exhaustion – and she subsequently wanted to leave the field. She interacted with a number of newly qualified medical doctors suffering the same experience. She fortunately did not leave the field due to burnout and managed to develop coping strategies with constant help. Her experience sparked interest in burnout and its consequences within newly qualified professionals employed in the South African hospital, specifically medical doctors. Furthermore, it highlighted the need to develop intervention strategies to prevent severe consequences to the field, the professionals and their private life, and/or society at large with the high numbers of beneficiaries. The research was approached with the assumption that burnout is present among newly qualified medical doctors working in that sector as there were reports of ongoing malfunctions in the system, and a high level of burnout was confirmed by the MBI-GS. It was also expected that burnout would impact various levels (personal, work, society, and beneficiaries). Member-checking was done to obtain the views of the findings and interpretations to establish credibility (Lincoln and Guba, 1985). The data was verified and scrutinized by the participants to ensure that it was a true reflection of their experiences. Detailed observation notes and reflection notes were kept to enhance dependability. Furthermore, a lengthy data collection process and interpretation of the data was undertaken; a full description was given of the setting, participants and methods; and a constant peer review process was established. Verbal statements, crucial pauses and overlaps were transcribed. Coding was applied to ensure reliability and the quality of the study based on phenomenological standards (Creswell, 2013). The findings were discussed taking the established theories and methods into account (Yin, 2018). Rigor was promoted through thick descriptions and transparent processes. The study provides an in-depth insight into the data, the findings and the topic, but does not provide generalizability since this is usually not in the scope of a qualitative study (Lincoln and Guba, 1985; Creswell, 2013).

## Findings

The findings are articulated in the following themes about the experience of the PP2.0 intervention for burnout among Generation Y medical doctors working in a South African public hospital Table 4 provides the overview of the themes and subthemes analyzed from the data set. The findings under the different subthemes respond directly to the overall research question: How do a sample of South African Generation Y medical doctors working in a South African public hospital understand and experience the intervention to combat burnout?

**TABLE 4:** Findings.

Overall theme	Subtheme
Opinions of the PP2.0 intervention	*Subtheme 1.1: Excitement at the idea of PP2.0 intervention.* *Subtheme 1.2: Lack of interest developed into appreciation.*
Alterations to the PP2.0 intervention	–
Benefits of the PP2.0 intervention	*Subtheme 3.1: Intimacy of the group.* *Subtheme 3.2: Comfortable group and one-on-one interaction.*
Lessons gained from the PP2.0 intervention	–
Implemented coping strategies	*Subtheme 5.1: Coping strategies expanded.* *Subtheme 5.2: Reframing of challenges and developing a positive outlook.*
Post-PP2.0 intervention experiences	*Subtheme 6.1: Failing health system and lack of support.* *Subtheme 6.2: Coping strategies continually under pressure.* *Subtheme 6.3: Need for ongoing PP2.0 intervention and debriefing sessions.* *Subtheme 6.4: Implemented coping strategies post-PP2.0 intervention.*

### Opinions of the PP2.0 intervention

The first theme was the general view of the PP2.0 intervention for burnout among Generation Y medical doctors. The main question posed was: “How did you feel about this PP2.0 intervention session?” The participants highlighted “excitement at the idea of intervention” and some “lack of interest developed into appreciation” as subthemes when describing their experiences of the intervention.

*Subtheme 1.1: Excitement at the idea of PP2.0 intervention*

Some participants welcomed the idea of PP2.0 intervention with the hope of sustainability since they feel they need constant PP2.0 intervention. This is indicated by the following remark by participant 1: “*Excitement I had about this intervention because my first year of working in the public hospital was terrible; I wish the intervention could happen more often*”; and participant 2 added, “*I’m hoping we get this sort of debriefing sessions regularly because we need them to cope.*” The other participants also perceived the PP2.0 intervention as an opportunity that was provided for self-reflection, which they deemed essential. It was seen in the following quotes: participant 5 stated, “*The MBI-GS questionnaire made one think and reflect. And to look within which I hardly do nowadays because of the work pressure.*” Participant 6 added, “*Even the intervention session created a need to introspect and reflect within which is important.*”

At the same time, they were cynical about whether stakeholders would respond positively and hear their pleas for better working conditions in the South African public hospital. They deemed that the findings would show they are overwhelmed and in need of help and emphasized that help is beneficial. Participant 10 said, “*I do not know whether the stakeholders would take the research to heart, they seem not to care about us.*” Another Generation Y medical doctor, participant 7, added, “*This research and intervention will show we are overwhelmed because of the poor working conditions and that we need help continually to cope, and how we benefited from your intervention, but I wonder if they will take it to heart.*”

*Subtheme 1.2: Lack of interest developed into appreciation*

Some participants were skeptical about the PP2.0 intervention session. The intern leader, who also attended, had reportedly pre-empted that the Generation Y medical doctors would not attend the PP2.0 intervention due to low morale; however, the intern leader encouraged them to attend. This came out during the PP2.0 intervention process. Voluntary participation was encouraged. Once the PP2.0 intervention session got underway, their previous reluctance turned into appreciation of the experience. Participant 6 said, “*Initially I was not looking forward to it, I came only because it was encouraged. I realize though I would have missed out if I stayed away.*” Participant 4 added, “*I imagined it will be a boring PowerPoint presentation. But glad it was not.*” Participant 3 reiterated, “*The idea of it being run by a clinical psychologist led to some weariness, because I have never been to a clinical psychologist. I am honestly pleasantly surprised by how it unfolded.*”

### Alterations to the PP2.0 intervention

The second theme highlighted was proposed changes or additions to the PP2.0 intervention. The main question posed was: “Is there anything that you wish could be changed or added to the PP2.0 intervention for burnout among Generation Y medical doctors?” All the participants in the phase II and III sessions perceived the PP2.0 intervention process for burnout among Generation Y medical doctors to be adequate. They were comfortable with the researcher’s approach and with the researcher as the coach. Participant 2 stated, “*Nothing really needs to be added or changed in the intervention process*”; participant 5 added, “*The approach of the intervention and you as the facilitator worked for me.*” Participants 1 and 8 stated, “*Can we do this again soon and keep all as was, in terms of numbers and the approach and you as the facilitator?*” and “*It felt informal and worked well for us.*”

### Benefits of the PP2.0 intervention

The third theme was the highlighted benefits of the PP2.0 intervention for burnout among Generation Y medical doctors. The question posed was: “Is there anything that stood out for you about the PP2.0 intervention and its approach?” The participants highlighted the intimacy of the group, the group being comfortable, and one-on-one interaction as subthemes when describing their experiences of the PP2.0 intervention for burnout among Generation Y medical doctors.

*Subtheme 3.1: Intimacy of the group*

There was an overall appreciation of the group size, which led to them being open, feeling heard and having shared experiences to normalize their experience. Participant 1 stated, “*The size was just perfect; not too big and overwhelming*”; and participant 3 added, “*I could be open because of the size of the group and the way you gave each person a chance to speak. I felt heard. I also felt like I am not going crazy because my colleagues are going through similar experiences.*”

*Subtheme 3.2: Comfortable group and one-on-one interaction*

There was a shared view that the interaction as a group with the facilitator was a good fit because they still received one-on-one attention. Each person was given a chance to speak. All the participants commented that they felt understood and listened to; and that the shared experiences led to them bonding as a team, normalizing their personal experiences and humanizing one another. They believed that a safe environment was created where they could be vulnerable in the presence of the others and yet be empowered to go forth. Participant 2 said, “*I felt we were all heard. Each of us was given a chance, not one talking over the other. It was not just you talking and we listening*”; participant 5 added, “*I felt safe and comfortable.*” Participant 6 stated, “*It made us reflect. It was not a formal presentation with boring PowerPoint slides.*” Participant 8 said, “*We got to know the others better in this – not just as a colleague*”; participant 7 added, “*It promoted cohesion for us as group and encouraged doing things outside the workplace as a group to relieve stress.*”

There was a general sense of normalization of the experiences of burnout and feelings of being supported and not being alone. Participant 3 said, “*I feel I am not going crazy or alone in experiencing negative emotions.*” There was a shared opinion that they could relate to the coach and that the approachability enhanced the interaction. Participant 1 said, “*Your openness and honesty, easy going manner was helpful for us*”; participant 4 added, “*You are relaxed and comfortable even about your experiences of burnout that made us comfortable.*” Participants 9 and 10 added, “*You made us feel safe and relaxed,”* and *“You were very professional in an ‘informal human’ manner.*”

### Lessons gained from the PP2.0 intervention

The fourth theme highlighted was about the lessons gained from the PP2.0 intervention. The main question posed was: “Is there anything that you can take with from today?” The overall view was that they significantly benefited from attending and that they wish for sustainable services of this nature. In support of this statement, participant 8 said, “*We truly gained from this experience, I wish there could be more sessions for support like this continually.*” There was verbalized empathy for colleagues who could not attend, but could also have benefited from the session. Participant 10 stated, “*The absent participants missed out because we really did benefit.*” There was an awareness of the indications of burnout, the potential ability to adjust, and the ability to refer others because of increased recognition of burnout presentations. Participant 3 said, “*I gained an increased awareness of depersonalization, emotional exhaustion and reduced professional competence manifestations in myself and my colleagues* (*including the seniors*)”; and participant 1 added, “*This awareness will create less personalization when attacked by senior doctors and conscious coping strategies in dealing with personal stress.*” Lastly, participant 5 noted that “*The junior medical doctors who are actually on sick leave really needed this. Thank you.*”

Participant 2 said, “*We as medical doctors always want to come across as having it together and that emotional things are not for us, because we are expected to cope as if we are not humans, thank you for reminding us that we are humans and it is ok*”, to which participant 4 added, “*Working with medical doctors who do not care is sad. Thank you for reminding us that it is ok to be different and still care.*” There was an unexpected benefit from the group intervention verbalized by the participants. Participant 9 said, *“It was a pleasant surprise how much value we got from this intervention*,” and participant 7 declared, “*We appreciate the leader of our group encouraging attendance, else we would have missed out on what we needed.*”

### Implemented coping strategies

The fifth theme highlighted was that the Generation Y medical doctors implemented coping strategies after being exposed to the PP2.0 intervention for burnout. The participants highlighted the expansion of their coping strategies. Reframing the challenges helped them to develop a positive outlook, which were the subthemes in describing their experiences of the PP2.0 intervention.

*Subtheme 5.1: Coping strategies expanded*

Coping strategies were expanded and the participants gained a better understanding of burnout and its impact on the self and others. There was also an awareness of required self-care activities. Participant 1 stated, “*This motivated me to care for myself even more*”; participant 2 added, “*I will develop more hobbies outside the medical field. I now have seen the need for self-reflection and have an increased awareness*”; and lastly, participant 3 stated that: “*When the intern group leader suggests activities outside of work, I will make a point of attending it, seeing it is a beneficial way to de-stress that we can adopt.*” There was a shared view of learnt burnout manifestation and related self-treatment, referral and self-care. Participant 6 said, “*I learned of secondary traumatization, which I often experience from hearing, seeing or working with trauma. I see now that I need to find a professional to vent out, not to traumatize family members/friends or pass on the trauma*”; while participant 5 stated, “*I need to take charge of my mind and perceptions. Additionally, my thoughts, emotions/feelings and actions/reactions; which is all I can take charge of and change.*”

Participant 4 noted, “*I know that I was called to do this, and I was reminded of this. The negative experiences I will not personalize and let them define me*”; participant 7 added, “*I will appreciate more attempts of the leader of our intern group who organizes certain events; and make more effort to attend.*” Participant 10 stated, “*I will identify a mentor and clinical psychologist to offload to regularly*”; and participant 9 said, “*I will use relaxation and breathing exercises I gathered here.*” Lastly, participant 8 stated, “*I need to talk more about my experiences at work instead of trying to process them alone, because then I end up snapping at everyone.*”

*Subtheme 5.2: Reframing of challenges and developing a positive outlook*

The participants gained the ability to reframe their challenges and develop a positive outlook. The Generation Y medical doctors verbalized a better understanding of senior doctors and potential ways to cope. Participant 1 stated, “*This awareness I gained from the intervention created less personalization when I feel attacked by senior doctors and conscious coping strategies in dealing with personal stress*”; and participant 2 stated, “*I understand that the environment, manager, health department and public sector difficulties are not in my control; therefore, I need to work on what I can control which is my thoughts, actions and emotions.*”

Participant 3 said, “*I understand that the failing system is not a reflection on me*”; participant 4 stated, “*The senior medical doctors themselves are burnt out, therefore we all need intervention*”; and lastly, participant 5 said, “*Going forth at least I understand they are burdened too and I would need to work on me personall*y.” There was an awareness about not being alone in this experience of burnout, which was emphasized by participant 6: “*I know I am not alone and what I feel is also felt by other junior medical doctors*,” and participant 7 stated, “*This session created cohesion in our group.*” Based on observation, the Generation Y medical doctors learnt to be change agents. This was suggested by participant 4: “*What I gathered from the session I gave to my fiancé and that helped him process some of the things he was going through*”; participant 8 added, “*I would not want to be like senior medical doctors going forth to the junior medical doctors if I am still in the public hospital.*”

### Post-PP2.0 intervention experiences

The sixth theme highlighted were their experiences after being exposed to the PP2.0 intervention of burnout among Generation Y medical doctors. The question posed was: “How are you doing post the PP2.0 intervention?”

*Subtheme 6.1: Failing health system and lack of support*

The participants seemed to be motivated to continue positively in the face of challenges in the public hospital. They were aware of the burnout experience and what is required to overcome it, which the public hospital system is unable to offer them. Therefore, they need to find ways to help them focus on themselves to survive.

Participant 1: “*The system is really failing us still, but I am still trying*”; participant 2 added, “*The support structures still need to be placed for us ASAP. I always have in mind and apply where possible what we discussed in the session.*” Participant 5 stated, “*Management needs to be more supportive. I struggle but at least I know now what I can control and that I am not alone and that it is not a reflection on me*”; to which participant 6 added, “*There is still a lack of or minimal support from hospital management and senior doctors. But post-intervention session we almost understand that it is like that because the system promoted this, and it is what they know therefore we need to focus on making sure I am able to constructively cope.*” Lastly, participant 7 stated, “*There still needs to be change in the public hospital and broader government in South Africa. I focus on what I can do and doing my best, not what I cannot change.*”

*Subtheme 6.2: Coping strategies continually under pressure*

There was a shared notion that even though they were ready to face challenges in the different rotations with a gained coping and reframing ability, the doctors’ coping strategies were constantly under pressure, especially in certain placements. Participant 9 mentioned that “*Some placements are harder than others still, but I have in the back of my mind coping strategies I need to access*”; participant 1 added, “*Some senior medical doctors are harder to handle than others, but I continually bear in mind that they are also struggling in a failing system*”; and lastly, participant 2 stated, “*I have on and off days. I wanted to specialize in pediatrics, but I see pediatrics consultants working longer hours than us, so I am not sure still. I am going to surgery and the call roster is not for us to influence, that is going to be a strenuous rotation. I can’t wait for it to go by.*”

*Subtheme 6.3: Need for ongoing intervention and debriefing sessions*

The participants continuously mentioned the need for sustainable and consistent support for coping; career path development; repeated interaction; and a relationship with a mentor to help with challenging cases. Participant 5 said, “*We need regular debriefing and support sessions for emotional/psychological beings*”; participant 9 declared, “*We need ongoing intervention to cope.*” Participant 1 stated, “*I need assistance to deal with stress regarding specialization. Because of the negative experiences in the public hospital, it takes away some of the pleasure in specializing in certain fields. I feel we need ongoing debriefing to choose a specialty objectively and to also not move away from the field because we are burnt out*”; and participant 2 said, “*When can we see you again, this was so helpful?*”

*Subtheme 6.4: Implemented coping strategies post*-*PP2.0 intervention*

Generation Y medical doctors mentioned that they implemented certain coping strategies after the PP2.0 intervention session, although they still experienced a high level of burnout, especially emotional exhaustion. The implemented coping strategies included self-care strategies, being aware of burnout, and applying coping strategies. There was a shared notion from the participants that they should start focusing on caring for themselves amid the difficult working conditions to prevent their burnout from becoming worse. They appeared to be empowered by the self-care strategies, even though they still needed more support in the long run and the conditions still had to change for the better.

Participant 1 said, “*I have some self-care strategies which I implemented: I am spacing out my calls, sleeping early, doing other things, meeting people and reflecting so that I am not on autopilot missing out on life’s precious moments.*” Participant 2 emphasized that they organized themselves to experience less strain: “*The public health system is still the same but working in flexible rotations where one can suggest or create a good environment for self-care like pediatrics made it easie*r.” Participant 5 added, “*Working with less severely ill patients made it easier to cope. For example, at pediatrics wards we were able to arrange that we start early and finish before 6 pm. Unlike oncology where I am always working with very sick patients, some dying.*”

The group indicated that they were more aware and had a better understanding of burnout; therefore, they could identify it in themselves, which makes it more manageable. A statement from participant 5 alluded to this: “*Indications of burnout I experienced before are now more manageable because I am able to identify them* (*have awareness of them*) *and I know I am not going crazy or alone.*” The awareness also made them more mindful of the coping strategies that work for them and those that exacerbate the negative experience as can be seen in the statement of participant 9: “*I have my days, but now I talk more about what I experienced at work instead of keeping it in. When do we see you again for our next session? Can we arrange one? This was so helpful.*”

The coping techniques include an ability to identify out-group connections/activities for minimal detachment to become energized between work times. Participant 7 stated, “*I am now meeting other people, making time to do other things outside of work and not with people from my work*,” to which participant 6 replied, “*I exercise and gym more; I am forcing myself to make time.*” They became potential change agents by using learnt awareness and experiences of self-care. Participant 5 said, “*I showed the notes to my husband when he was going through something and it helped him process some of the things*,” to which participant 7 added, “*I will be able to identify burnout and suggest to someone to seek help.*” Lastly, participant 1 said, “*I am more understanding that the senior medical doctors are also under pressure, experience burnout, though it does not excuse some of their behavior. I wish they could see the need to seek help and for stakeholders to assist everyone in this regard.*”

There will be a need for ongoing PP2.0 intervention for burnout among Generation Y medical doctors to maintain the ability to cope to some extent when working in the public hospital.

Findings show that Generation Y medical doctors are challenged within the South African context due to challenges on micro-, meso-, and macro-levels of culture and structural aspects. Findings further show which specific coping strategies the participants have developed within their socio-cultural context. Some culture-specific aspects are highlighted in the discussion.

## Discussion

The study presents ten Generation Y medical doctors’ burnout experiences and six of their post- PP2.0 intervention experiences of working in a local hospital that is part of the South Africa’s public health care sector. As highlighted in the limitation section, the discussion only presents the views of the participants of the study. In summary, the findings point to a need for ongoing PP2.0 intervention for burnout in the limited sample, and appreciation for the PP2.0 intervention received. This study could open a discussion for a bigger sample and further research into an empirically based PP2.0 intervention for the at-risk population.

The development of burnout is gradual, and it can isolate individuals even if they experience it in a team. However, solidarity is only felt when members of the group realize that they all have similar burnout experiences and weaknesses (Bährer-Kohler, 2013), as the participants of this study experienced during the intervention. Additionally, psychological interventions in the South African context are often stigmatized. It takes courage to admit that there is a problem, to seek help, to do some self-reflection, and to recognize your own vulnerability (Vogel et al., 2006). The Generation Y medical doctors were aware of their difficulties, yet they could not articulate their experience; they did not receive the expected support in their workplace and believed that they were expected to cope, even using maladjusted coping strategies. In the PP2.0 intervention they were able to better understand burnout, identify it in their seniors, and have increased awareness of the impact of burnout. Furthermore, the need to work on their personal beneficial coping strategies and changing internal views of the workplace was encouraged during the PP2.0 intervention.

According to the literature (Cox, 2013), interventions should be customized to the clients’ needs, like in the case of the Generation Y medical doctors. During the PP2.0 intervention, active listening and empathy were used as methods and the doctors felt heard and understood. The facilitator emphasized a humanistic paradigm and its qualities, such as unconditional positive regard and reflection of feelings, which promoted a sense of being heard (Rogers, 1959). The working hypothesis developed in this study, based on the intervention experience is *A customized intervention for burnout among Generation Y medical doctors where they feel heard, understood, have shared experiences, and understand their own experiences will promote intrinsic growth.*

For the Generation Y medical practitioners, opening up in group PP2.0 interventions needs trustful relationships initiated by the facilitator, prioritizing the needs of the clients. Their needs such as a comfortable group size, one-on-one discussions, and rapport with the facilitator, were met. According to the feedback from the participants, certain lessons, benefits and skills were gained from the PP2.0 intervention, which were according to the clients’ needs. The clients’ openness led to the overall learning of skills and an appreciation of the benefits of PP2.0 interventions. Being heard and understood in a PP2.0 intervention environment was a catharsis, support and normalization, which, in turn, promoted group cohesion and common hope (Rogers, 1959; Mason, 2002; Finlay, 2008), and contributed to an overall positive experience for the participants.

Overall investment in employees’ mental health and burnout prevention will give a return on investment for employers (Maslach et al., 2001; Bauer et al., 2003; Walter et al., 2013; Ruiz, 2019). The experience of the PP2.0 intervention promoted participants’ expansion of their coping strategies, reframed challenges and developed positive outlooks. Generally, suggestions for burnout intervention in literature include a need to focus on a combination of self, organizational and situational factors (Felton, 1998). Prevention and intervention strategies were individual, or group-directed, focused on the organization; or a combination of both (Walter et al., 2013). In general, an integrative interdisciplinary approach to burnout was followed that incorporates prevention, medical, psycho-educational and communication promotion in the South African health care context. The intervention is classified as a PP2.0 intervention since it addresses on the one hand the factors of burnout and helps the participants on the other hand to get into a PP space to work constructively and positively with the experiences.

This study focused on individual PP2.0 intervention in a specific small group and did not aim to change the public hospital system. The study rather aimed to empower them to cope with challenging situations. Post the PP2.0 intervention, the participants were still challenged as their workplace was still the same. However, they felt better prepared to cope with the situation. Therefore, the individual focus of the intervention seemed to benefit individual and group-oriented levels. At the same time, the benefits from the PP2.0 intervention for burnout are likely to be short-lived, because they are constantly under pressure. They might experience even more pressure from their life challenges; therefore, ongoing intervention sessions for debriefing in future are essential for them and are highly recommended. However, special enrichment was the time spent with the facilitator who listened to them. The PP2.0 intervention session may only be a starting point, but it initiated a personal awareness and consciousness of how to increase coping skills. It is the first step toward developing long-term coping strategies and empowerment to take decisions.

## Conclusion

The PP2.0 intervention benefited the participants: they gained an increased self-knowledge of burnout, insight into their seniors and peers, and coping skills. However, the benefits could be short-lived due to the poor working conditions and the individual’s burnout experience. The participants are always under pressure and might experience other stressors as they are facing the challenges of life. This could challenge the skills they gained. Therefore, there is a need for continuous PP2.0 intervention sessions and restructuring of parts of the local hospital. The phenomenological and qualitative approach followed during the PP2.0 intervention sessions made the participants feel understood, comfortable and open to the intervention process; thus, leading them to gain from the process. The PP2.0 intervention was non-threatening, informal, allowed for self-reflection and gained from a good match of the facilitator and the client to be successful. The following limitations should be noted for the study. All analyses are interpreted very cautiously, given the underpowered design. Also, the sample size is limited.

### Recommendations for future research and practice, the public hospital, and consultants and facilitators

In terms of theoretical contributions, previous studies suggested that burnout should be treated as an organization-wide problem (Panagioti et al., 2016; Lemaire and Wallace, 2017). This is also reflected in the findings and should be investigated in larger samples in the same at-risk population – the Generation Y medical doctors working in the public hospital.

Future studies could also focus on exploring the differences between Generation Y and other generations; and should also include more diverse groups of health professionals who could benefit from PP2.0 interventions, such as nurses. Since this study primarily included black people from various ethnic groups, future studies should include individuals from other South African cultural groups.

On a practical note, future studies should also aim to intervene at all three levels: the individual, the group and the organization. They should specifically take emotional exhaustion and new burnout developments into consideration.

Furthermore, consultants and facilitators need to develop awareness of generation-specific needs and develop PP2.0 interventions and strategies accordingly. The consultants and facilitators should be empathetic; act on the needs of the clients; be approachable and interactive; and strive for internal and external growth.

## Data availability statement

The raw data supporting the conclusions of this article will be made available by the authors, without undue reservation.

## Ethics statement

The studies involving human participants were reviewed and approved by University of South Africa and Polokwane Mankweng Hospital Complex, Department of Health Limpopo Province. The research was conducted in an ethical manner bounded by the ethical code of psychology in South Africa (Health Professions Council of South Africa). The patients/participants provided their written informed consent to participate in this study.

## Author contributions

RO and C-HM wrote and edited the manuscript. KD conducted the research and wrote the manuscript. All authors contributed to the article and approved the submitted version.
